# CT Diagnosis of Thyroid Hemiagenesis Following Inconclusive Ultrasound in the Absence of Nuclear Medicine Imaging

**DOI:** 10.7759/cureus.108704

**Published:** 2026-05-12

**Authors:** Abisiniya Kabtimer, Nitsuh Ayele, Abera Girma

**Affiliations:** 1 Surgery, Wolkite University Hospital, Wolkite, ETH; 2 Orthopedics, St. Paul’s Millennium Medical College, Addis Ababa, ETH

**Keywords:** case report, computed tomography, ethiopia, hemiagenesis, hemithyroid, inconclusive ultrasound, resource-limited setting

## Abstract

Thyroid hemiagenesis (THA) is a rare congenital condition in which one lobe of the thyroid fails to develop and is often discovered incidentally during imaging for unrelated conditions. We report the case of a 21-year-old woman who presented with generalized fatigue, cold intolerance, and irregular menstrual cycles. Physical examination and laboratory findings, including thyroid function tests, were within normal limits.

An initial neck ultrasound was inconclusive due to technical limitations and the inadequate visualization of the thyroid anatomy. A subsequent computed tomography (CT) scan demonstrated the absence of the left thyroid lobe, confirming the diagnosis of thyroid hemiagenesis. Due to a lack of access to nuclear medicine imaging, radionuclide scanning was not performed. The patient was managed conservatively and advised regular follow-up. Thyroid hemiagenesis is often underdiagnosed and may be associated with other endocrine abnormalities, although many patients remain clinically stable.

This case highlights the importance of adapting imaging strategies by utilizing available modalities when initial studies are inconclusive and gold standard investigations are not accessible. To our knowledge, this is the first documented case of thyroid hemiagenesis in Ethiopia and underscores the importance of awareness and flexibility in the diagnostic approach to rare congenital thyroid anomalies.

## Introduction

Thyroid hemiagenesis (THA) is a rare congenital condition in which one lobe of the thyroid gland fails to develop, with an estimated prevalence of 0.05%-0.5% [1]. It exhibits a female predilection and most commonly involves the left lobe [2]. The majority of affected individuals are asymptomatic and euthyroid, leading to underdiagnosis and underreporting. Most cases are discovered incidentally during imaging for unrelated indications.

Embryologically, the thyroid gland originates from the foramen cecum at the base of the tongue and descends through the thyroglossal duct to its final pretracheal position, where it bifurcates into two lateral lobes connected by the isthmus. Disruption during this developmental process can result in various congenital anomalies, including hemiagenesis, ectopic thyroid tissue, or thyroglossal duct cysts [3]. While the exact etiology remains unclear, genetic factors involving transcription factors such as TTF1, TTF2, and PAX8 have been implicated, with familial cases reported in monozygotic twins [4].

Despite its often-asymptomatic presentation, thyroid hemiagenesis is clinically important due to its association with compensatory thyroid changes and an increased risk of nodular and autoimmune thyroid diseases such as Graves’ disease and Hashimoto’s thyroiditis [5]. Although most cases are diagnosed incidentally in euthyroid patients, the recognition of this entity is important as it may influence future surveillance strategies, clinical decision-making, and patient counseling, particularly in scenarios where thyroid pathology develops, and surgical intervention or lifelong hormone replacement may be required [6].

Thyroid scintigraphy using technetium-99m or iodine-123 is considered the gold standard for the functional confirmation of thyroid hemiagenesis, as it assesses thyroid tissue uptake and identifies absent or ectopic functioning tissue. Ultrasound is the first-line imaging modality for anatomical evaluation due to its availability and safety, although it is operator-dependent. Computed tomography (CT) may serve as an additional anatomical tool when ultrasound is inconclusive, but it does not provide functional assessment and cannot replace scintigraphy [7]. Furthermore, thyroid hemiagenesis may remain asymptomatic until it becomes clinically significant, at which point diagnostic evaluation, including blood tests, can reveal associated thyroid pathology such as Graves’ disease [8,9].

In resource-limited settings where nuclear medicine is unavailable, clinicians must rely on alternative imaging modalities to establish the diagnosis. To date, no case of thyroid hemiagenesis has been reported in Ethiopia. We present the first documented case, diagnosed by computed tomography following an inconclusive ultrasound, and propose a diagnostic algorithm applicable to similar resource-constrained environments.

## Case presentation

A 21-year-old female patient presented to the outpatient department at Wolkite Specialized Hospital in September 2025 with a three-month history of generalized fatigue, cold intolerance, and irregular menstrual cycles. She had no known chronic medical illnesses and no history of neck swelling, pain, dysphagia, or hoarseness. There was no family history of thyroid disease or other hereditary disorders. She had no prior surgical history and was not taking any medications.

On physical examination, the patient was clinically euthyroid and hemodynamically stable. Vital signs were within normal limits. There was no conjunctival pallor, exophthalmos, or lid lag. Neck examination revealed no palpable masses, thyromegaly, cervical lymphadenopathy, or surgical scars.

Thyroid function tests, including thyroid-stimulating hormone (TSH), free T3, and free T4, were within normal limits (Table 1). Complete blood count, basic metabolic panel, and serum calcium were within normal ranges (Tables 1-3).

**Table 1:** Thyroid Function Test Results at Presentation Thyroid function tests, including thyroid-stimulating hormone (TSH), free T3, and free T4, were within normal limits. Reference ranges are provided for each parameter. Values indicate a euthyroid state at the time of presentation

Test	Value	Reference Range
TSH	2.1 µIU/mL	0.4-4.5
Free T3	3.2 pg/mL	2.3-4.2
Free T4	1.1 ng/dL	0.8-1.8

**Table 2:** Complete Blood Count (CBC) Findings CBC values were within normal reference ranges

Test	Result	Reference Range
White Blood Cell (WBC)	5.8 × 10³/µL	4.0-11.0 × 10³/µL
Red Blood Cell (RBC) Count	4.9 × 10⁶/µL	4.2-5.9 × 10⁶/µL
Hemoglobin (Hb)	12.0 g/dL	12.0-16.0 g/dL
Hematocrit (Hct)	37%	36%-46%
Mean Corpuscular Volume (MCV)	90 fL	80-100 fL
Platelet Count (PLT)	351 × 10³/µL	150-450 × 10³/µL

**Table 3:** Basic Metabolic Panel and Electrolyte Findings Serum electrolytes and metabolic parameters were within normal reference ranges

Test	Result	Reference Range
Sodium (Na)	138 mmol/L	135-145 mmol/L
Potassium (K)	4.1 mmol/L	3.5-5.0 mmol/L
Magnesium (Mg)	2.0 mg/dL	1.7-2.2 mg/dL
Phosphate (PO₄)	3.6 mg/dL	2.5-4.5 mg/dL
Calcium (Ca)	9.0 mg/dL	8.5-10.5 mg/dL

Initial imaging

The initial neck ultrasound was limited by technical factors and suboptimal acoustic windows, which resulted in the incomplete visualization of the thyroid gland. The right thyroid lobe was partially visualized and appeared grossly normal in echotexture; however, detailed assessment was limited. The isthmus was not reliably identified, and the left thyroid lobe could not be adequately visualized due to acoustic shadowing and patient-related factors, preventing the definitive anatomical assessment of the full thyroid gland. As a result, the study was deemed inconclusive, and further cross-sectional imaging with CT was pursued for anatomical confirmation.

Advanced imaging

Given the inconclusive ultrasound and the unavailability of nuclear medicine services, a computed tomography (CT) scan of the neck was obtained. The scan extended from the skull base to the thoracic inlet, allowing the evaluation of the thyroid bed and adjacent cervical structures; no ectopic thyroid tissue was identified within the imaged field, although the dedicated assessment of the lingual thyroid region and mediastinum was not performed. Both non-contrast and contrast-enhanced phases were acquired to provide comprehensive anatomical evaluation, with the non-contrast phase facilitating the identification of intrinsic thyroid tissue and the contrast-enhanced phase improving the delineation of vascular anatomy and confirming the presence or absence of enhancing thyroid tissue. In this young patient, radiation exposure and iodinated contrast use were carefully considered, and CT was performed only after inconclusive ultrasound and in the absence of radionuclide scintigraphy to achieve a definitive diagnosis while minimizing unnecessary repeat imaging.

Imaging findings

Non-contrast CT

Non-contrast axial CT of the neck demonstrates a well-defined right thyroid lobe in its expected anatomical location. No identifiable thyroid tissue is visualized in the left thyroid bed. The trachea is midline without significant deviation. The left internal jugular vein and surrounding cervical vascular structures are normally positioned.

Contrast-Enhanced CT

**Figure 1:**
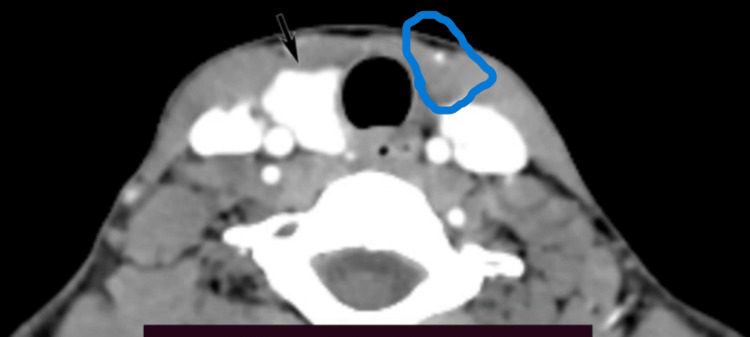
Axial Contrast-Enhanced CT Scan of the Neck Axial contrast-enhanced CT scan of the neck demonstrating the absence of the left thyroid lobe, with the blue line encircling the empty thyroid bed. The arrow indicates the right thyroid lobe, which is present CT: computed tomography

**Figure 2:**
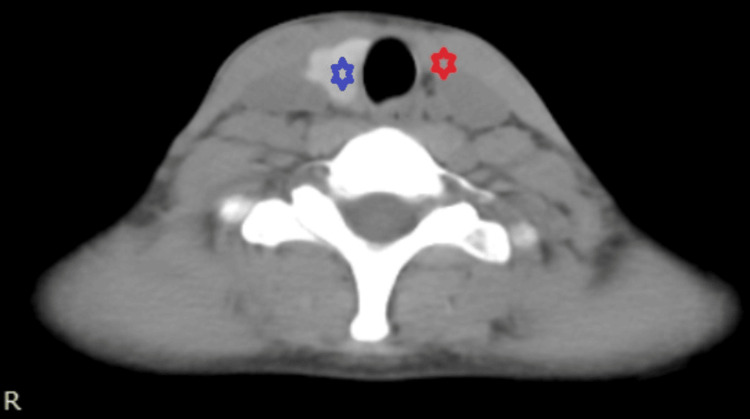
Axial Non-contrast CT Scan of the Neck Demonstrating Thyroid Hemiagenesis With the Absence of the Left Thyroid Lobe Axial non-contrast CT of the neck showing a normal right thyroid lobe, homogeneous hyperdense structure (blue star) and the absence of the left thyroid lobe with an empty left thyroid bed (red star), consistent with thyroid hemiagenesis CT: computed tomography

**Figure 3:**
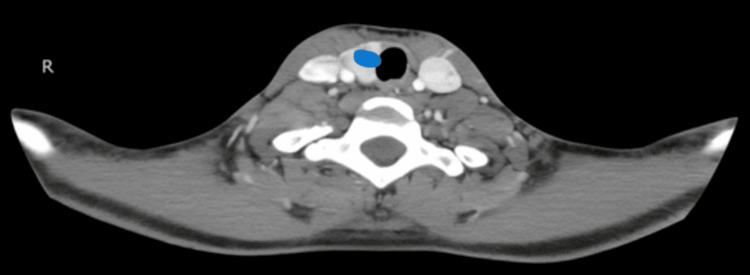
Axial Contrast-Enhanced CT Scan of the Neck Axial contrast-enhanced CT scan of the neck showing a normally enhancing right thyroid lobe, blue dot (the left side of the image corresponds to the patient’s right), with no identifiable thyroid tissue in the contralateral (left) thyroid bed CT: computed tomography

Contrast-enhanced axial CT images demonstrate the homogeneous enhancement of the right thyroid lobe. The left thyroid lobe remains absent, with no evidence of ectopic thyroid tissue within the visualized neck region. No focal mass, calcification, or cervical lymphadenopathy is identified (Figures 1-3).

Diagnosis

The imaging findings are consistent with the congenital absence of the left thyroid lobe, compatible with thyroid hemiagenesis.

Management

As the patient was clinically and biochemically euthyroid, no active medical or surgical intervention was required, and no follow-up imaging was performed. The patient was counseled regarding the benign, congenital nature of the condition and reassured, with advice for annual clinical evaluation and thyroid function testing to monitor for potential late-onset dysfunction.

## Discussion

Thyroid hemiagenesis (THA) is a rare congenital anomaly with a low reported prevalence in population studies, contributing to its classification as an uncommon thyroid disorder [6]. Many affected individuals remain clinically euthyroid and asymptomatic, which leads to underdiagnosis and incidental detection when imaging is performed for unrelated indications [6]. Although most cases occur sporadically, genetic factors involved in thyroid development, including transcription factors such as TTF1, TTF2, and PAX8, have been implicated in thyroid dysgenesis [3,6].

Adults with thyroid hemiagenesis often remain euthyroid; however, the solitary lobe may undergo compensatory hypertrophy or be predisposed to nodular changes and autoimmune thyroid disease [6,7]. Previous reports have also demonstrated that thyroid hemiagenesis may remain clinically silent until associated with other thyroid pathology, emphasizing the importance of thorough clinical and imaging evaluation in symptomatic individuals [5,8].

Ultrasound is widely regarded as the first-line and most accessible tool for thyroid evaluation due to its noninvasive nature and ease of use. However, its diagnostic accuracy is operator-dependent and may be limited by technical factors such as the poor visualization of the thyroid anatomy, patient body habitus, and equipment constraints [7]. In this case, the initial neck ultrasound was inconclusive because the left thyroid lobe could not be visualized adequately, illustrating a scenario in which first-line imaging alone was insufficient.

When first-line imaging, such as ultrasound, is inconclusive and nuclear medicine modalities are not available, cross-sectional imaging such as CT can provide detailed anatomical information to help confirm the diagnosis of thyroid hemiagenesis [7,8]. Radionuclide scintigraphy remains the functional imaging standard for confirming the absence of thyroid tissue in suspected agenesis; however, access to nuclear medicine may be unavailable in many resource-limited settings, as in our patient’s context [1,8]. Although CT provides valuable anatomical information, it has limitations compared to radionuclide scintigraphy, particularly its inability to assess thyroid function; nevertheless, in this case, it served as an adaptive imaging modality, offering the necessary anatomical confirmation and guiding clinical management in the absence of nuclear medicine services.

This case underscores the necessity of adapting imaging strategies to the resources at hand, demonstrating that a combination of clinical judgment and the appropriate use of available imaging modalities can lead to accurate diagnosis even in settings with limited access to specialized imaging.

A literature search of PubMed and Google Scholar did not identify any previously published reports of thyroid hemiagenesis from Ethiopia at the time of manuscript preparation. To our knowledge, this is the first documented case of thyroid hemiagenesis in Ethiopia and adds to the growing literature on the variable presentations and diagnostic pathways of this rare entity.

## Conclusions

To our knowledge, this is the first reported case of thyroid hemiagenesis documented in Ethiopia. This case highlights the importance of recognizing congenital thyroid anomalies, particularly in resource-limited settings where such conditions may be underreported. It also underscores the role of CT imaging as a useful anatomical diagnostic tool when nuclear medicine is not available, although it does not provide the functional assessment of thyroid tissue. Overall, this case demonstrates that diagnosis can be achieved through the adaptive use of available imaging modalities, supported by careful clinical and radiological correlation.
